# Nurse educators’ satisfaction with online Objective Structured Clinical Examination scoring system *

**DOI:** 10.1590/1518-8345.6816.4344

**Published:** 2024-11-15

**Authors:** Fahni Haris, Ferika Indarwati, Yanuar Primanda, Resti Yulianti Sutrisno, Kellyana Irawati, Yin-Hwa Shih

**Affiliations:** 1 Universitas Muhammadiyah Yogyakarta, School of Nursing, Bantul, Yogyakarta, Indonesia.; 2 Asia University, Departement of Healthcare Administration, Wufeng, Taichung, Taiwan.; 3 Queensland University of Technology, School of Nursing, Brisbane, Queensland, Australia.

**Keywords:** Nursing Education, Educational Technology, Internet, Participant Satisfaction, Questionnaires, Developing Nations

## Abstract

the paper-based OSCE (Pa-OSCE) scoring system has several drawbacks, including significant paper waste and being time-consuming. This study aims to assess examiner satisfaction with the On-OSCE scoring management system, identify and weigh the potential benefits of the innovation, and promote its use.

using a cross-sectional study design, we developed satisfaction inventory consisting of four domains: time-saving, user-friendliness, prospective application, and objectivity. This inventory was used to compare satisfaction scores between two examiner groups (a total of 67 subjects).

the 20-item satisfaction inventory demonstrated high validity and reliability (0.98 and 0.97, respectively). The average scoring time was significantly shorter in the online scoring management system group (p<0.001). Both the total satisfaction score and the scores for each domain were significantly higher in the online scoring management system group compared to the paper-based scoring management system group (p<0.001).

a high proportion of the examiners provided positive feedback on the online scoring management system. The online scoring management system saves the time to score, and is more objective, easier to use, and is recommended for implementation. Online scoring management systems can be implemented in nursing education institutions globally to improve scoring efficiency. Additionally, the 20-item satisfaction inventory can serve as a benchmarking tool to assess educators worldwide.

## Highlights

**(1)** Valid and reliable 20-item questions on the nursing scoring system.

**(2)** A high proportion of the examiners (lecturers) provided positive feedback on the online OSCE.

**(3)** Nursing education institutions may adopt the On-OSCE scoring system to improve scoring.

**(4)** The utilization of On-OSCE can save time, objective, and simplify the scoring process.

**(5)** The On-OSCE scoring system is highly recommended for worldwide implementation.

## Introduction

 The objective structured clinical examination (OSCE) was developed in the United Kingdom to assess medical students’ clinical competencies in a controlled, simulated environment ^(^ 1 ^)^ . The OSCE has been introduced and used to assess students’ clinical competencies in various health professional education programs, including nursing ^(^ 2 ^-^ 4 ^)^ . There are several clinical competencies assessment methods in health professional education, such as the mini clinical evaluation exercise (mini-CEX) and direct observation of procedural skills (DOPS); however, OSCE remains the most widely used method for health professional qualification examinations, including in Asian countries like Indonesia ^(^ 5 ^-^ 7 ^)^ . 

 The OSCE examination has several test stations with simulated environments ^(^ 7 ^-^ 9 ^)^ , and during the exam, students are monitored and evaluated by an examiner using predetermined marking criteria ^(^ 10 ^)^ . The OSCE reduces examiner bias, standardizes the exam procedure, and provides objectivity to evaluate clinical competencies ^(^ 1 ^)^ . Moreover, the OSCE highlights the students’ strengths and weaknesses ^(^ 11 ^-^ 13 ^)^ , assesses their knowledge and attitudes towards clinical practice ^(^ 14 ^)^ , provides profound learning ^(^ 4 ^)^ , and builds students’ self-confidence ^(^ 3 ^,^ 5 ^)^ . Both students and examiners have acknowledged the satisfaction and benefits of the OSCE experience in their education ^(^ 2 ^,^ 15 ^-^ 17 ^)^ . Furthermore, the OSCE has received excellent feedback as an assessment tool for evaluating clinical competence and addressing student diversity in education ^(^ 12 ^,^ 18 ^)^ . Interestingly, there are significant limitations associated with OSCEs. Some students find them stressful, and they require considerable resources, including clinical skills laboratories, equipment, and examiners ^(^ 13 ^)^ . Despite the challenges, authors believe that the educational benefits of OSCEs significantly outweigh the concerns related to resource allocation ^(^ 16 ^)^ . 

 OSCE scoring management system in health professions and nursing education has traditionally been conducted using a paper-based method (Pa-OSCE). However, examiners have reported several issues with the Pa-OSCE system, including lost assessment sheets, missing details such as students numbers and names, and illegible handwriting ^(^ 19 ^)^ . Additionally, OSCE scores are not immediately available, which creates time pressure and reduces efficiency in resource and management of OSCE examiners ^(^ 20 ^)^ . Our previous study confirmed the concerns associated with Pa-OSCE, including significant paper waste, excessive time spent by examiners manually computing the score based on the checklist, and the checklist’s absence of an appropriate feedback column for evaluating examinee performance ^(^ 21 ^)^ . Therefore, developing an online OSCE (On-OSCE) scoring management system is essential, particularly in Indonesian nursing education institutions, to address these gaps in the Pa-OSCE ^(^ 22 ^)^ . 

 Several studies have evaluated assessors’ satisfaction with the implementation of the online OSCE scoring management system ^(^ 13 ^,^ 16 ^,^ 23 ^)^ . These studies have explored examiners’ perceptions and attitudes toward the online scoring management system in nursing education in Ireland ^(^ 23 ^)^ . The findings indicated high levels of examiner satisfaction. The online OSCE scoring management system reduced the missing data and improved the provision of timely feedback to students ^(^ 23 ^)^ . Similarly, a study on the online assessment of OSCE in pharmacist education institutions in Taiwan reported that examiners demonstrated high recognition, acceptance, and satisfaction with the online assessment system ^(^ 24 ^)^ . Additionally, the online OSCE scoring management system facilitates data storage and enables detailed analysis of overall group or individual students performance, which supports further teaching and learning evaluation ^(^ 23 ^)^ . 

 Although the online OSCE scoring management system is well-established in developed countries ^(^ 3 ^,^ 6 ^,^ 23 ^)^ , its implementation in developing countries such as Indonesia represents a novel approach, particularly in nursing education. Our previous study revealed that the common OSCE scoring management system in Indonesia relied on traditional paper-based scoring management system (Pa-OSCE) ^(^ 25 ^)^ . To address issues such as excessive paper waste and time consumption, our nursing department has developed an online scoring system (On-OSCE) ^(^ 21 ^)^ . To ensure the effectiveness and acceptance of the On-OSCE among administrators and examiners accustomed to paper-based assessment techniques, it is crucial to evaluate its practical use. Thus, this study aims to assess examiner satisfaction with the On-OSCE scoring management system, identify, and weigh the potential benefits of this innovation, and promote its adoption. 

## Method

### Study design

This study utilized a cross-sectional approach, wherein the investigator simultaneously measures both the outcomes and the participants’ exposures. The study was conducted at the School of Nursing, Universitas Muhammadiyah Yogyakarta, Indonesia, from June 21, 2019 to February 28, 2021. Data collection spanned three years to support the transition from a paper-based OSCE to an online OSCE scoring management system, and to enable data capture from both groups (paper-based vs. online).

### Participants

We used a total sampling technique in this study. A total of 30 examiners using a paper-based OSCE scoring management system (Pa-OSCE) and 37 examiners using an online OSCE scoring management system (On-OSCE) participated in the study. The examiners were nursing lecturers or lecturer assistants from the School of Nursing, Universitas Muhammadiyah Yogyakarta, Indonesia, who regularly conduct OSCE assessments at the nursing school.

### Instruments

 Before collecting the data, we developed a satisfaction inventory containing 20 items spanning four domains: time-saving (6 items), user-friendliness (5 items), prospective application (4 items), and objectivity (5 items), as detailed in Table 1 . The subjects were asked to rate their satisfaction with each item on an 11-point Likert-type scale ranging from unsatisfied (0) to highly satisfied (10) ^(^ 26 ^)^ . The instrument was developed by researchers based on the literature review on this topic. We categorized subjects’ satisfaction into three groups based on their total score; 0-70 indicated *low satisfaction* , 71-140 indicated *medium satisfaction* , and 141-200 indicated *high satisfaction* . 

 We recruited five faculty members with experience in Pa-OSCE and On-OSCE to conduct an inventory content validity index (CVI). The CVI consists of content suitable (CVIs) and word precise (CVIw) ^(^ 27 ^)^ . The CVI scores of five experts were 0.98. Three or more experts with an average CVI of 0.78 or higher are considered to have good content validity ^(^ 28 ^)^ . We also examined the item-total correlation value across the four domains (a total of 20 questions) to assess examiners’ satisfaction using the Pa-OSCE and the On-OSCE scoring management system. Items with a correlation value greater than 0.3 were retained. The Composite Reliability (CR) for the extreme was statistically significant ( *p* < 0.05). 

**Table 1 -:** The item analysis of the satisfaction inventory. Bantul, Yogyakarta, Indonesia, 2019-2021

**Question**	**Item**	Extreme group CR* value	**Item-total correlation**	**Note**
1	Time-saving	14.11 ^†^	0.97	keep
2		19.95 ^†^	0.96	keep
3		17.71 ^†^	0.97	keep
4		20.11 ^†^	0.96	keep
5		23.90 ^†^	0.98	keep
6		13.47 ^†^	0.96	keep
1	User-friendliness	6.66 ^†^	0.92	keep
2		8.84 ^†^	0.95	keep
3		9.36 ^†^	0.95	keep
4		12.25 ^†^	0.94	keep
5		9.99 ^†^	0.87	keep
1	Prospective application	7.71 ^†^	0.97	keep
2		8.01 ^†^	0.97	keep
3		7.42 ^†^	0.92	keep
4		9.44 ^†^	0.91	keep
1	Objectivity	7.64 ^†^	0.71	keep
2		10.03 ^†^	0.80	keep
3		6.71 ^†^	0.72	keep
4		3.17 ^†^	0.39	keep
5		10.13 ^†^	0.58	keep

*CR Composite reliability

^†^ Significant value p < 0.001

 The reliability (Cronbach’s α) and internal consistency (comparisons of extreme groups and corrected item-total correlation) were analyzed using the data from 15 examiners who conducted the Pa-OSCE scoring management system and 15 examiners who conducted the On-OSCE scoring management system. Cronbach’s α score was 0.97. With an alpha > 0.60, the questionnaire can be considered reliable ^(^ 26 ^,^ 29 ^)^ . 

### Data collection

Research invitations were sent via WhatsApp application to all eligible participants. Participants who expressed willingness to participate in the study could contact the researchers if further information related to the research were needed. The survey of examiners’ satisfaction was administered online using Google Forms. The Google Forms included 20 questions covering four variables (time-saving, user-friendliness, prospective application, and objectivity). Completing and submitting the Google forms indicated participants’ consent to participate in this study.

### Data analysis

The quantitative data obtained from examiner performance at OSCE stations and the questionnaire survey were analyzed descriptively using Microsoft Excel 365 for Windows 10 (Microsoft Corporation, Redmond, WA, USA) and IBM SPSS statistics version 22 statistical software (IBM Corporation, Armonk, NY, USA). Descriptive data were presented in frequency and percentage for categorical data such as participants’ gender, education, and teaching experience, or mean for continuous variables such as participants’ age. Mean rank was used to describe data in minutes to finish the scoring and satisfaction scores in four domains (time-saving, user-friendliness, prospective application, and objectivity).

 Further analysis was conducted to ascertain differences between the Pa-OSCE and On-OSCE groups in demographics, the time required to complete students’ scores, and satisfaction from examiners’ perspectives. Appropriate tests, such as Fisher’s Exact, Chi-square, or Mann-Whitney U tests, were used to investigate mean differences between the paper-based and the online OSCE scoring groups on the four examiners’ satisfaction domains. Data for total examiners’ satisfaction scores were initially categorized into three categories (low, medium, and high). However, due to the very few responses in the low satisfaction category, this group was merged with the medium satisfaction category to enable meaningful analysis. Statistical significance was defined as *p* < 0.05. 

### Ethical considerations

Ethical clearance was approved by the Ethical Committee of the Faculty of Medicine and Health Sciences, Universitas Muhammadiyah Yogyakarta (no. 051/EC-KEPK FKIK UMY/II/2019). Participants were informed about the study’s goal and provided verbal consent to participate after being fully informed. They were assured that their data remained confidential. Responses were kept anonymous, and a non-disclosure agreement was established, giving the respondents the right to withdraw from the study at any time.

## Results

### Demographic data of the participants

Table 2 shows statistical differences between groups based on the categories of age, gender, educational level, and teaching experience. The result shows that participants’ characteristics in both groups were quite similar, except for age. The category of age was significantly different among these four variables, while gender, education level, and teaching experience were not significant ( *p* > 0.05). The mean age of respondents was 34.10 ± 5.58 in the Pa-OSCE group and 30.70 ± 5.78 in the On-OSCE group ( *p* = 0.011). Most examiners were female, in both the Pa-OSCE group (90%) or the On-OSCE group (89.19%). Most examiners held graduate degree levels, with 66.67% in the Pa-OSCE group and 56.76% in the On-OSCE group. The respondents’ teaching experience was categorized as less than or equal to one year experienced and less than ten years experienced. 

**Table 2 -:** Demographic characteristics of respondents. Bantul, Yogyakarta, Indonesia, 2019-2021

**Variable**	Pa-OSCE* N (%)	**On-OSCE** ^†^ **N (%)**	***p* -Value**
Gender			1.000 ^‡^
Female	27 (90.00)	33 (89.19)	
Male	3 (10.00)	4 (10.81)	
Educational level			0.458 ^‡^
Undergraduate	10 (33.33)	16 (43.24)	
Graduate	20 (66.67)	21 (56.76)	
Teaching experience			0.732 ^‡^
≤ 1 year	12 (40.00)	13 (35.14)	
≤ 5 years	6 (20.00)	12 (32.43)	
≤ 10 years	10 (33.33)	10 (27.03)	
> 10 years	2 (6.67)	2 (5.40)	
Age [Mean (SD ^§^ )]	34.10 (5.58)	30.70 (5.78)	0.011 ^||^

*Pa-OSCE Paper-based OSCE (objective structured clinical examination) scoring management system group;

**^†^ On-OSCE:** Computer-based OSCE (objective structured clinical examination) scoring management system group;

^‡^ Fisher’s Exact test;

^§^ SD Standard Deviation;

^||^ Mann-Whitney test

### Examiners spent less time scoring with On-OSCE

 There was a statistically significant difference in time required to finish the scoring OSCE in Pa-OSCE and On-OSCE. The mean rank of the On-OSCE group was lower than Pa-OSCE group, as shown in Table 3 . Pa-OSCE examiners spent more time calculating the score, whereas the scores in the On-OSCE were calculated automatically by the system. The data reveal that examiners saved more time in assigning the final score to the student in the On-OSCE group. 

**Table 3 -:** The mean rank of average time spent on scoring between the two groups using the Mann-Whitney U test. Bantul, Yogyakarta, Indonesia, 2019-2021

Pa-OSCE*	**On-OSCE** ^†^	***p-* Value**
Time spent on scoring	49.58	21.36	< 0.001

*Pa-OSCE Paper-based OSCE (objective structured clinical examination) scoring management system group;

**^†^On-OSCE:** Computer-based OSCE (objective structured clinical examination) scoring management system group

### The examiners had positive feedback on the On-OSCE scoring management system

 Satisfaction scores in four domains of the inventory are shown in Table 4 . A statistically significant difference was reported between the groups. The results indicated that the On-OSCE offered time-saving, user-friendliness, prospective applications, and objectivity compared to the Pa-OSCE. On-OSCE improved scoring efficiency (time-saving) and objectivity and was accepted (user-friendliness) for future use (prospective applications) by the majority of examiners. 

**Table 4 -:** The mean rank of satisfaction scores in four domains using the Mann-Whitney U test. Bantul, Yogyakarta, Indonesia, 2019-2021

**Variable**	Pa-OSCE*	**On-OSCE** ^†^	***p* -Value**
Time-saving (0 - 60)	16.30	48.35	<0.001
User-friendliness (0 - 50)	18.48	46.58	<0.001
Prospective application (0 - 40)	15.62	48.91	<0.001
Objectivity (0 - 50)	22.80	43.08	<0.001
Total satisfaction score	15.82	48.74	<0.001

*Pa-OSCE Paper-based OSCE (objective structured clinical examination) scoring management system group;

**^†^On-OSCE:** Computer-based OSCE (objective structured clinical examination) scoring management system group

### Examiners’ satisfaction was higher in the On-OSCE group compared to the Pa-OSCE group

 We combined the low and medium total satisfaction scores categories for statistical reasons. Examiners’ satisfaction with Pa-OSCE and On-OSCE is shown in Table 5 . A high proportion of On-OSCE examiners (97.30%) were highly satisfied with scoring students’ skills, while a high proportion of Pa-OSCE examiners (86.67%) reported low satisfaction levels. Tables 3 and 4 demonstrate that On-OSCE improved scoring efficiency and objectivity, with a high proportion of the examiners expressing satisfaction with the new scoring management system. 

**Table 5 -:** The satisfaction level of Pa-OSCE and On-OSCE using Fisher’s exact test. Bantul, Yogyakarta, Indonesia, 2019-2021

**Satisfaction level (total scores)**	Pa-OSCE* N (%)	**On-OSCE** ^†^ **N (%)**	***p-* Value /Cramer’s V** ^‡^
High satisfaction (141 – 200)	4 (13.33)	36 (97.30)	< 0.001/0.85
Low to medium satisfaction (0 – 140)	26 (86.67)	1 (2.70)	
Total	30 (100)	37 (100)	

*Pa-OSCE Paper-based OSCE (objective structured clinical examination) scoring management system group;

**^†^ On-OSCE:** Computer-based OSCE (objective structured clinical examination) scoring management system group;

**^‡^ Cramer’s V:** SPSS analysis to gain the association between variables (Pa-OSCE and On-OSCE)

## Discussion

 This study aims to comprehensively assess examiners’ satisfaction with the On-OSCE scoring management system, leveraging validated tools developed through factor analysis. The evaluation was conducted across four key aspects (timesaving, user-friendliness, prospective application, and objective) with a high validity and reliability inventory. Moreover, this study conclusively demonstrated that examiners were able to save significant time when assigning final scores to students within the On-OSCE scoring management system ( Table 3 ). Among four key aspects, the On-OSCE group showed high satisfaction ( Table 4 ). The results showed a high proportion (97.30%) of the examiners were satisfied with the efficiency (timesaving) and objectiveness of the scoring and accepted for future application (user-friendliness and prospective application) compared to the Pa-OSCE ( *p* < 0.001) ( Table 5 ). 

 One of the most heartbreaking findings in our context was the dramatic improvement in time finishing score; to calculate the final score and to decide whether the examinee passed or failed on the skill being examined. The study showed that by employing the On-OSCE scoring management system, examiners required less time to complete the scoring process than the Pa-OSCE scoring management system ( Table 3 ). Our hypothesis was coherent with several studies indicating that the online OSCE scoring management system reduced the official result faster than the traditional OSCE scoring management system ^(^ 22 ^,^ 30 ^)^ . There are four reasons why the On-OSCE scoring management system was faster than the Pa-OSCE scoring management system. First, the On-OSCE scoring management system calculated students’ scores automatically, ensured a non-missing checklist was submitted, marked easily, and had no handwriting needed. Second, the online OSCE scoring management system (tablet or PC) allows examiners to mark the checklist easily, effortlessly, and continuously ^(^ 10 ^)^ . Additionally, the marked checklist by the examiners is processed automatically by the system, causing students’ scores to appear immediately ^(^ 24 ^)^ . Third, the On-OSCE scoring management system prompts against missing marks by the systems. As with the alarm system, the examiner receives an alert on the tablet or PC screen if a mark is missing ^(^ 24 ^)^ . Fourth, the On-OSCE scoring management system grants examiners a straightforward spot on the checklist ^(^ 7 ^,^ 22 ^)^ . The examiners easily “click” the On-OSCE checklist instead of marking it by pen on the Pa-OSCE scoring management system. Examiners merely use the On-OSCE scoring management system by flipping over the checklist sheet by dragging or scrolling the checklist on the screen ^(^ 30 ^-^ 31 ^)^ . Thus, the On-OSCE scoring management system was superior to the Pa-OSCE scoring management system in completing student assessments. These findings believe that the application would be beneficial for nursing students and nursing institutions as our prior studies stated that students were satisfied with being assessed by the On-OSCE scoring management system, as they retrieved their scores immediately and their scores were incredibly accurate ^(^ 15 ^)^ . Additionally, we assessed the readiness of nursing education in Indonesia for implementing the On-OSCE scoring management system and hence acknowledged that most of them were ready to realize this application in their institution ^(^ 25 ^)^ . 

 Assessing examiners’ satisfaction with the implementation of the On-OSCE scoring management system is vital to gain an understanding, particularly of its feasibility, and provide fundamental information for further improvement programs. Our results showed that examiners were highly satisfied with giving students’ scores using the On-OSCE scoring management system (97.30%) compared to the Pa-OSCE scoring management system, which conveyed low satisfaction (86.67%) ( Table 5 ). Regarding the utilization of online OSCE scoring management systems, examiners assessed their satisfaction level in four domains: time savings, user-friendliness, prospective applications, and objectives ( Table 4 ). Other studies discovered that electronic tools facilitated the analysis of aggregated results, resulting in significant time savings ^(^ 13 ^,^ 30 ^)^ . Similarly, utilizing our unique electronic system significantly reduced the time required for data analysis, providing additional time for data interpretation to improve curriculum development and the evaluation of the learning process which might be one of the reasons why this scoring management system satisfied examiners. Furthermore, the On-OSCE scoring management system became an effortless future application for examiners since they were quickly recognized and familiar with the system; examiners reported being highly satisfied in all four domains. However, the authors stated that examiners must obtain adequate training and practice to be confident in using the computerized assessment system ^(^ 24 ^)^ . 

 According to the findings of this study, it was realized that there were three major reasons for the examiners’ satisfaction with utilizing the On-OSCE scoring management system. First, they might sign or create comments on the assessment form at any moment, which they could not do with the paper-based scoring management system. The online OSCE scoring management system was designed so examiners could effectively provide their feedback adequately. This finding aligns with previous research indicating that online OSCE offers a viable means to evaluate students’ competencies and provide immediate feedback on assessed abilities ^(^ 11 ^,^ 22 ^)^ . Second, the On-OSCE scoring management system disabled the add/subtract score function, making it easier for the examiners to objectively give the students’ overall scores according to their performance. Adding or subtracting the students’ scores is usually done by examiners in the Pa-OSCE scoring management system, decreasing the scoring objectivity. Third, Table 2 shows that there is a statistically significant difference in age between examiners. Comparing the ages, the On-OSCE group is younger than the Pa-OSCE group. It might be because young adult examiners have experience in digital competencies such as computer use, internet services ^(^ 32 ^)^ , and being exposed to better technological developments compared to older people ^(^ 33 ^)^ . Therefore, future work should be considered, especially to analyze factors that affect examiners’ decisions in the electronic OSCE assessment system. 

 During the COVID-19 pandemic, the On-OSCE scoring management system allows examiners to examine students’ skills without face-to-face assessment ^(^ 31 ^)^ . Moreover, a study acknowledged that students’ favorable response to the modified online OSCE scoring management system supports an online assessment to enhance the traditional OSCE scoring management system ^(^ 7 ^)^ . However, students believed that the On-OSCE scoring management system makes examiners overlook their behaviors because they focus on laptops or tablets, which may increase students’ anxiety ^(^ 15 ^)^ . Furthermore, the On-OSCE scoring management system can be utilized in conjunction with the traditional OSCE scoring management system as long as institutions have an adequate internet connection, appropriate hardware (tablet or PC), legitimate skill checklists, and appropriately trained examiners ^(^ 21 ^)^ . 

 Clinical proficiency is examined consistently, comprehensively, and organized on a wide spectrum. The examination highly demands an objective process in which the examiner’s bias must be diminished, and the discrimination between students’ performance levels must be shown ^(^ 22 ^)^ . The hypothesis was coherent with studies showing that the online OSCE scoring management system has reduced the administrative issue ^(^ 6 ^,^ 30 ^)^ . Moreover, our results, consistent with the study, revealed that the electronic software saved time and eliminated the possibility of missing data ^(^ 23 ^)^ . In addition, the potential benefits of electronic software include the ability to store, and analyze aggregate and individual findings, and provide students with immediate objective feedback ^(^ 23 ^)^ . 

 This study still has limitations. First, as a single-site study, the findings may not generalize to other institutions, nations, or healthcare specialties. Second, we did not assess examiners’ perceptions of the amount of mental effort necessary. The experienced examiners’ mental effort may affect their performance while observing and evaluating the students, which might affect the examiners’ satisfaction level. Thus, future work may be arranged to investigate the examiners’ mental effort on Pa-OSCE and On-OSCE. Third, our study variables and sample size are limited and do not meet the statistical requirements or assumptions required to run the logistic regression test ^(^ 34 ^)^ . Therefore, future research needs to be conducted to assess variables that may contribute to students’ and examiners’ satisfaction comprehensively and improve sample sizes to enable regression tests. 

## Conclusion

The On-OSCE scoring management system can alleviate the challenges encountered during the rigorous OSCE process. In comparison to the traditional OSCE scoring management system, the On-OSCE scoring management system offers the advantages of improved time-saving, user-friendliness, prospective application, and objectivity in assessing students’ performance. Examiners who are familiar with the system’s functionality demonstrated higher satisfaction with overall function and system utilization than the Pa-OSCE scoring management system. The On-OSCE scoring management system enhances the effectiveness of planning and implementation of the OSCE. It has tremendous advantages and is viable for clinical practice testing, which could be widely employed in practical learning in connected medical domains in the future. Moreover, further studies may use the recent 20-item satisfaction inventory to assess nurse educators worldwide.
