# Supplementary material for: Nurse educators’ satisfaction with online Objective Structured Clinical Examination scoring system
Source: Rev Lat Am Enfermagem. 2024 Nov 15;32:e4344. doi: 10.1590/1518-8345.6816.4344 (PMC11653744; doi:10.1590/1518-8345.6816.4344)
Supplement: Translation

# Translation: Satisfacción de los educadores de enfermería con el sistema de puntuación del Objective Structured Clinical Examination en línea *

**DOI:** 10.1590/1518-8345.6816.4343

**Published:** 2024-11-15

**Authors:** Fahni Haris, Ferika Indarwati, Yanuar Primanda, Resti Yulianti Sutrisno, Kellyana Irawati, Yin-Hwa Shih

**Affiliations:** ^1^ Universitas Muhammadiyah Yogyakarta, School of Nursing, Bantul, Yogyakarta, Indonesia.; ^2^ Asia University, Departement of Healthcare Administration, Wufeng, Taichung, Taiwan.; ^3^ Queensland University of Technology, School of Nursing, Brisbane, Queensland, Australia.

**Keywords:** Educación en Enfermería, Tecnología Educacional, Internet, Satisfacción del Participante, Cuestionarios, Países en Desarollo

## Abstract

el sistema de puntuación del OSCE en papel (Pa-OSCE) tiene varias desventajas, incluyendo un desperdicio significativo de papel y el hecho de que consume mucho tiempo. Este estudio tiene como objetivo evaluar la satisfacción de los examinadores con el sistema de gestión de puntuación del On-OSCE, identificar y evaluar los posibles beneficios de la innovación y promover su uso.

usando un diseño de estudio transversal, desarrollamos un inventario de satisfacción que consiste en cuatro dominios: ahorro de tiempo, facilidad de uso, aplicación prospectiva y objetividad. Este inventario fue utilizado para comparar las puntuaciones de satisfacción entre dos grupos de examinadores (un total de 67 individuos).

el inventario de satisfacción de 20 ítems demostró alta validez y confiabilidad (0,98 y 0,97, respectivamente). El tiempo medio de puntuación fue significativamente menor en el grupo del sistema de gestión de puntuación en línea (p<0,001). Tanto la puntuación total de satisfacción como las puntuaciones de cada dominio fueron significativamente mayores en el grupo del sistema de gestión de puntuación en línea en comparación con el grupo del sistema de gestión de puntuación en papel (p<0,001).

una gran proporción de los examinadores proporcionó *feedback* positivo sobre el sistema de gestión de puntuación en línea. El sistema de gestión de puntuación en línea ahorra tiempo en la puntuación, es más objetivo, más fácil de usar y se recomienda para su implementación. Los sistemas de gestión de puntuación en línea pueden ser implementados en instituciones de enseñanza de enfermería en todo el mundo para mejorar la eficiencia en la puntuación. Además, el inventario de satisfacción de 20 ítems puede servir como una herramienta de referencia para evaluar a los educadores en todo el mundo.

## Destacados

**(1)** Preguntas de 20 ítems válidas y confiables sobre el sistema de puntuación de enfermería.

**(2)** Una alta proporción de examinadores (profesores) proporcionó *feedback* positivo sobre el OSCE en línea.

**(3)** Las instituciones de enseñanza de enfermería pueden adoptar el sistema de puntuación del On-OSCE para mejorar la evaluación.

**(4)** La utilización del On-OSCE puede ahorrar tiempo, ser objetiva y simplificar el proceso de puntuación.

**(5)** El sistema de puntuación del On-OSCE es altamente recomendado para su implementación a nivel mundial.

## Introducción

 El Examen Clínico Objetivo Estructurado (OSCE) fue desarrollado en el Reino Unido para evaluar las competencias clínicas de los estudiantes de medicina en un entorno controlado y simulado ^(^ 1 ^)^ . El OSCE fue introducido y utilizado para evaluar las competencias clínicas de los estudiantes en varios programas de educación profesional en el área de la salud, incluida la enfermería ^(^ 2 ^-^ 4 ^)^ . Existen varios métodos de evaluación de competencias clínicas en la educación de profesionales de la salud, como el mini-ejercicio de evaluación clínica (mini-CEX) y la observación directa de habilidades procedimentales (DOPS); sin embargo, el OSCE sigue siendo el método más utilizado para exámenes de calificación de profesionales de la salud, incluso en países asiáticos como Indonesia ^(^ 5 ^-^ 7 ^)^ . 

 El examen OSCE cuenta con varias estaciones de prueba con entornos simulados ^(^ 7 ^-^ 9 ^)^ y, durante el examen, los estudiantes son monitoreados y evaluados por un examinador utilizando criterios de puntuación predefinidos ^(^ 10 ^)^ . El OSCE reduce el sesgo del examinador, estandariza el procedimiento del examen y proporciona objetividad para evaluar las competencias clínicas ^(^ 1 ^)^ . Además, el OSCE destaca los puntos fuertes y débiles de los estudiantes ^(^ 11 ^-^ 13 ^)^ , evalúa sus conocimientos y actitudes en relación con la práctica clínica ^(^ 14 ^)^ , promueve un aprendizaje profundo ^(^ 4 ^)^ y aumenta la confianza en sí mismos de los estudiantes ^(^ 3 ^,^ 5 ^)^ . Tanto los estudiantes como los examinadores reconocieron la satisfacción y los beneficios de la experiencia del OSCE en su formación ^(^ 2 ^,^ 15 ^-^ 17 ^)^ . Además, el OSCE ha recibido excelentes comentarios como herramienta de evaluación de la competencia clínica y de la diversidad estudiantil en la educación ^(^ 12 ^,^ 18 ^)^ . Cabe destacar que existen limitaciones significativas asociadas con los OSCE. Algunos estudiantes los consideran estresantes y requieren recursos considerables, incluidos laboratorios de habilidades clínicas, equipos y examinadores ^(^ 13 ^)^ . A pesar de los desafíos, autores creen que los beneficios educativos de los OSCE superan significativamente las preocupaciones relacionadas con la asignación de recursos ^(^ 16 ^)^ . 

 El sistema de gestión de puntuación OSCE en las profesiones de la salud y la educación en enfermería se ha llevado a cabo tradicionalmente mediante un método basado en papel (Pa-OSCE). Sin embargo, los examinadores han informado de varios problemas con el sistema Pa-OSCE, como la pérdida de hojas de evaluación, la falta de detalles, como los números y nombres de los estudiantes, y la caligrafía ilegible ^(^ 19 ^)^ . Además, las puntuaciones del OSCE no están disponibles de inmediato, lo que genera presión de tiempo y reduce la eficiencia de los recursos y la gestión de los examinadores del OSCE ^(^ 20 ^)^ . Nuestro estudio anterior confirmó las preocupaciones asociadas con el Pa-OSCE, como el desperdicio significativo de papel, el tiempo excesivo que los examinadores dedican a calcular manualmente la puntuación basada en la lista de verificación y la falta de una columna adecuada para *feedback* en la lista de verificación para evaluar el desempeño del estudiante ^(^ 21 ^)^ . Por lo tanto, el desarrollo de un sistema de gestión de puntuación del OSCE en línea (On-OSCE) es esencial, especialmente en las instituciones de enseñanza de enfermería de Indonesia, para abordar estas deficiencias del Pa-OSCE ^(^ 22 ^)^ . 

 Varios estudios han evaluado la satisfacción de los evaluadores con la implementación del sistema de gestión de puntuación del OSCE en línea ^(^ 13 ^,^ 16 ^,^ 23 ^)^ . Estos estudios exploraron las percepciones y actitudes de los examinadores hacia el sistema de gestión de puntuación en línea en la enseñanza de enfermería en Irlanda ^(^ 23 ^)^ . Los resultados indicaron altos niveles de satisfacción entre los examinadores. El sistema de gestión de puntuación del OSCE en línea redujo la falta de datos y mejoró la entrega oportuna de *feedback* a los estudiantes ^(^ 23 ^)^ . De manera similar, un estudio sobre la evaluación en línea del OSCE en instituciones de enseñanza de farmacia en Taiwán informó que los examinadores mostraron un alto reconocimiento, aceptación y satisfacción con el sistema de evaluación en línea ^(^ 24 ^)^ . Además, el sistema de gestión de puntuación del OSCE en línea facilita el almacenamiento de datos y permite un análisis detallado del rendimiento general del grupo o de cada estudiante, lo que contribuye a una evaluación más profunda de la enseñanza y el aprendizaje ^(^ 23 ^)^ . 

 Aunque el sistema de gestión de puntuación del OSCE en línea está bien establecido en países desarrollados ^(^ 3 ^,^ 6 ^,^ 23 ^)^ , su implementación en países en desarrollo, como Indonesia, representa un nuevo enfoque, especialmente en la enseñanza de enfermería. Nuestro estudio anterior reveló que el sistema común de gestión de puntuación del OSCE en Indonesia se basaba en el sistema tradicional de gestión de puntuación basado en papel (Pa-OSCE) ^(^ 25 ^)^ . Para resolver problemas como el desperdicio excesivo de papel y el consumo de tiempo, nuestro departamento de enfermería desarrolló un sistema de puntuación en línea (On-OSCE) ^(^ 21 ^)^ . Para garantizar la eficacia y aceptación del On-OSCE entre los administradores y examinadores acostumbrados a las técnicas de evaluación basadas en papel, es fundamental evaluar su uso práctico. Por lo tanto, este estudio tiene como objetivo evaluar la satisfacción de los examinadores con el sistema de gestión de puntuación del On-OSCE, identificar y sopesar los posibles beneficios de esta innovación y promover su adopción. 

## Métodos

### Diseño del estudio

 Este estudio utilizó un enfoque transversal, en el cual el investigador mide simultáneamente los resultados y las exposiciones de los participantes. El estudio se llevó a cabo en la Escuela de Enfermería de la *Universitas Muhammadiyah Yogyakarta* , Indonesia, del 21 de junio de 2019 al 28 de febrero de 2021. La recolección de datos se extendió durante tres años para apoyar la transición de un OSCE en papel a un sistema de gestión de puntuación de OSCE en línea y permitir la captura de datos de ambos grupos (en papel vs. en línea). 

### Participantes

 Utilizamos una técnica de muestreo total en este estudio. Un total de 30 examinadores utilizando un sistema de gestión de puntuación de OSCE en papel (Pa-OSCE) y 37 examinadores utilizando un sistema de gestión de puntuación de OSCE en línea (On-OSCE) participaron en el estudio. Los examinadores eran profesores de enfermería o asistentes de profesores de la Escuela de Enfermería de la *Universitas Muhammadiyah Yogyakarta* , Indonesia, que realizaban regularmente evaluaciones OSCE en la escuela de enfermería. 

### Instrumentos

 Antes de recolectar los datos, desarrollamos un inventario de satisfacción con 20 ítems que abarcaba cuatro dominios: ahorro de tiempo (6 ítems), facilidad de uso (5 ítems), aplicación prospectiva (4 ítems) y objetividad (5 ítems), como se detalla en la Tabla 1 . Se solicitó a los participantes que clasificaran su satisfacción con cada ítem en una escala tipo Likert de 11 puntos, que iba desde insatisfecho (0) hasta muy satisfecho (10) ^(^ 26 ^)^ . El instrumento fue desarrollado por los investigadores con base en la revisión de la literatura sobre este tema. Clasificamos la satisfacción de los participantes en tres grupos según su puntuación total: 0-70 indicaba baja satisfacción, 71-140 indicaba satisfacción media y 141-200 indicaba alta satisfacción. 

 Reclutamos a cinco miembros del cuerpo docente con experiencia en Pa-OSCE y On-OSCE para llevar a cabo un índice de validez de contenido del inventario (IVC). El IVC consiste en contenido adecuado (IVCs) y precisión de palabras (IVCw) ^(^ 27 ^)^ . Las puntuaciones del IVC de cinco expertos fueron de 0,98. Tres o más expertos con un IVC promedio de 0,78 o más se consideran que tienen buena validez de contenido ^(^ 28 ^)^ . También examinamos el valor de la correlación ítem-total en los cuatro dominios (un total de 20 preguntas) para evaluar la satisfacción de los examinadores con el uso del Pa-OSCE y del sistema de gestión de puntuación On-OSCE. Se mantuvieron los ítems con un valor de correlación mayor de 0,3. La Confiabilidad Compuesta (CC) para el extremo fue estadísticamente significativa (p < 0,05). 

**Tabla 1 -:** El análisis de ítems del inventario de satisfacción. Bantul, Yogyakarta, Indonesia, 2019-2021

**Pregunta**	**Ítem**	Grupo extremo Valor CC*	**Correlación ítem-total**	**Nota**
1	Ahorro de tiempo	14,11 ^†^	0,97	mantener
2		19,95 ^†^	0,96	mantener
3		17,71 ^†^	0,97	mantener
4		20,11 ^†^	0,96	mantener
5		23,90 ^†^	0,98	mantener
6		13,47 ^†^	0,96	mantener
1	Facilidad de uso	6,66 ^†^	0,92	mantener
2		8,84 ^†^	0,95	mantener
3		9,36 ^†^	0,95	mantener
4		12,25 ^†^	0,94	mantener
5		9,99 ^†^	0,87	mantener
1	Aplicación prospectiva	7,71 ^†^	0,97	mantener
2		8,01 ^†^	0,97	mantener
3		7,42 ^†^	0,92	mantener
4		9,44 ^†^	0,91	mantener
1	Objetividad	7,64 ^†^	0,71	mantener
2		10,03 ^†^	0,80	mantener
3		6,71 ^†^	0,72	mantener
4		3,17 ^†^	0,39	mantener
5		10,13 ^†^	0,58	mantener

*CC Confiabilidad compuesta

^†^ Valor significativo p < 0,001

 La confiabilidad (α de Cronbach) y la consistencia interna (comparaciones de grupos extremos y correlación ítem-total corregida) fueron analizadas utilizando los datos de 15 examinadores que realizaron el sistema de gestión de puntuación Pa-OSCE y 15 examinadores que realizaron el sistema de gestión de puntuación On-OSCE. El puntaje α de Cronbach fue de 0,97. Con un alfa > 0,60, el cuestionario puede considerarse confiable ^(^ 26 ^,^ 29 ^)^ . 

### Recolección de datos

 Las invitaciones para la encuesta se enviaron por medio de la aplicación *WhatsApp* a todos los participantes elegibles. Los participantes que expresaron su disposición para participar en el estudio podían ponerse en contacto con los investigadores si necesitaban más información relacionada con la investigación. La encuesta de satisfacción de los examinadores se administró en línea utilizando *Google Forms* . Los formularios de *Google* incluían 20 preguntas que abarcaban cuatro variables (ahorro de tiempo, facilidad de uso, aplicación prospectiva y objetividad). El llenado y envío de los formularios indicaba el consentimiento de los participantes para participar en este estudio. 

### Análisis de datos

 Los datos cuantitativos obtenidos del desempeño de los examinadores en las estaciones del OSCE y de la encuesta mediante cuestionarios se analizaron de manera descriptiva utilizando *Microsoft Excel 365* para *Windows 10* (Microsoft Corporation, Redmond, WA, EE.UU.) y el *software* estadístico *IBM SPSS Statistics* versión 22 (IBM Corporation, Armonk, NY, EE.UU.). Los datos descriptivos se presentaron en frecuencia y porcentaje para datos categóricos, como género, formación y experiencia docente de los participantes, o media para variables continuas, como la edad de los participantes. La media de clasificación se utilizó para describir los datos en minutos para completar la puntuación y las puntuaciones de satisfacción en cuatro dominios (ahorro de tiempo, facilidad de uso, aplicación prospectiva y objetividad). 

 Se realizó un análisis más profundo para verificar las diferencias entre los grupos Pa-OSCE y On-OSCE en términos demográficos, el tiempo necesario para completar las puntuaciones de los estudiantes y la satisfacción desde la perspectiva de los examinadores. Se utilizaron pruebas apropiadas, como las pruebas exactas de Fisher, Chi **-** cuadrado o Mann **-** Whitney U, para investigar las diferencias promedio entre los grupos de puntuación del OSCE en papel y en línea en los cuatro dominios de satisfacción de los examinadores. Los datos de las puntuaciones totales de satisfacción de los examinadores se categorizaron inicialmente en tres categorías (baja, media y alta). Sin embargo, debido a las pocas respuestas en la categoría de baja satisfacción, este grupo se fusionó con la categoría de satisfacción media para permitir un análisis significativo. La significancia estadística se definió como p < 0,05. 

### Consideraciones éticas

 La autorización ética fue aprobada por el Comité de Ética de la Facultad de Medicina y Ciencias de la Salud de la *Universitas Muhammadiyah Yogyakarta* (nº 051/EC-KEPK FKIK UMY/II/2019). Se informó a los participantes sobre el objetivo del estudio y dieron su consentimiento verbal para participar después de ser completamente informados. Se les aseguró que sus datos se mantendrían confidenciales. Las respuestas se mantuvieron anónimas y se estableció un acuerdo de no divulgación, otorgando a los encuestados el derecho de retirarse del estudio en cualquier momento. 

## Resultados

### Datos demográficos de los participantes

 La Tabla 2 muestra las diferencias estadísticas entre los grupos según las categorías de edad, género, nivel educativo y experiencia docente. El resultado muestra que las características de los participantes en ambos grupos eran bastante similares, con la excepción de la edad. La categoría de edad fue significativamente diferente entre estas cuatro variables, mientras que el género, el nivel educativo y la experiencia docente no fueron significativos (p > 0,05). La edad promedio de los encuestados fue de 34,10 ± 5,58 en el grupo Pa-OSCE y de 30,70 ± 5,78 en el grupo On-OSCE (p = 0,011). La mayoría de los examinadores eran mujeres, tanto en el grupo Pa-OSCE (90%) como en el grupo On-OSCE (89,19%). La mayoría de los examinadores tenía nivel de posgrado, con un 66,67% en el grupo Pa-OSCE y un 56,76% en el grupo On-OSCE. La experiencia docente de los encuestados se categorizó en menos de un año o igual a un año de experiencia y menos de diez años de experiencia. 

**Tabla 2 -:** Características demográficas de los encuestados. Bantul, Yogyakarta, Indonesia, 2019-2021

**Variables**	Pa-OSCE* N (%)	**On-OSCE** ^†^ **N (%)**	**Valor de p**
Sexo			1,000 ^‡^
Femenino	27 (90,00)	33 (89,19)	
Masculino	3 (10,00)	4 (10,81)	
Nivel educativo			0,458 ^‡^
Grado	10 (33,33)	16 (43,24)	
Posgrado	20 (66,67)	21 (56,76)	
Experiencia docente			0,732 ^‡^
≤ 1 año	12 (40,00)	13 (35,14)	
≤ 5 años	6 (20,00)	12 (32,43)	
≤ 10 años	10 (33,33)	10 (27,03)	
> 10 años	2 (6,67)	2 (5,40)	
Edad [Media (SD ^§^ )]	34,10 (5,58)	30,70 (5,78)	0,011 ^||^

*Pa-OSCE Grupo del sistema de gestión de puntuación del OSCE (examen clínico estructurado objetivo) basado en papel;

^†^ On-OSCE = Grupo del sistema de gestión de puntuación del OSCE (examen clínico estructurado objetivo) basado en línea;

^‡^ Prueba exacta de Fisher;

^§^ SD Desviación estándar;

^||^ Prueba de Mann-Whitney

### Los examinadores pasaron menos tiempo puntuando con el On-OSCE

 Hubo una diferencia estadísticamente significativa en el tiempo necesario para completar la puntuación del OSCE entre el Pa-OSCE y el On-OSCE. La clasificación media del grupo On-OSCE fue menor que la del grupo Pa-OSCE, como muestra la Tabla 3 . Los examinadores del Pa-OSCE pasaron más tiempo calculando la puntuación, mientras que las puntuaciones en el On-OSCE fueron calculadas automáticamente por el sistema. Los datos revelan que los examinadores ahorraron más tiempo al asignar la puntuación final a los estudiantes del grupo On-OSCE. 

**Tabla 3 -:** Clasificación media del tiempo promedio dedicado a la puntuación entre los dos grupos utilizando la prueba U de Mann-Whitney. Bantul, Yogyakarta, Indonesia, 2019-2021

Pa-OSCE*	**On-OSCE** ^†^	**Valor de p**
Tiempo dedicado a la puntuación	49,58	21,36	< 0,001

*Pa-OSCE: Grupo del sistema de gestión de puntuación del OSCE (examen clínico estructurado objetivo) basado en papel;

**^†^ On-OSCE:** Grupo del sistema de gestión de puntuación del OSCE (examen clínico estructurado objetivo) basado en línea

###  Los examinadores tuvieron un *feedback* positivo sobre el sistema de gestión de puntuación On-OSCE 

 Las puntuaciones de satisfacción en cuatro dominios del inventario se muestran en la Tabla 4 . Se registró una diferencia estadísticamente significativa entre los grupos. Los resultados indicaron que el On-OSCE ofreció ahorro de tiempo, facilidad de uso, aplicaciones prospectivas y objetividad en comparación con el Pa-OSCE. El On-OSCE mejoró la eficiencia de la puntuación (ahorro de tiempo) y la objetividad, y fue aceptado (fácil de usar) para uso futuro (aplicaciones prospectivas) por la mayoría de los examinadores. 

**Tabla 4 -:** Clasificación media de las puntuaciones de satisfacción en cuatro dominios utilizando la prueba U de Mann-Whitney. Bantul, Yogyakarta, Indonesia, 2019-2021

**Variables**	Pa-OSCE*	**On-OSCE** ^†^	**Valor de p**
Ahorro de tiempo (0 - 60)	16,30	48,35	<0,001
Facilidad de uso (0 - 50)	18,48	46,58	<0,001
Aplicación prospectiva (0 - 40)	15,62	48,91	<0,001
Objetividad (0 - 50)	22,80	43,08	<0,001
Índice de satisfacción total	15,82	48,74	<0,001

*Pa-OSCE: Grupo del sistema de gestión de puntuación del OSCE (examen clínico estructurado objetivo) basado en papel;

**^†^ On-OSCE :**Grupo del sistema de gestión de puntuación del OSCE (examen clínico estructurado objetivo) basado en línea

### La satisfacción de los examinadores fue mayor en el grupo On-OSCE en comparación con el grupo Pa-OSCE

 Combinamos las categorías de puntuaciones de satisfacción total bajas y medias por motivos estadísticos. La satisfacción de los examinadores con el Pa-OSCE y el On-OSCE se muestra en la Tabla 5 . Una gran proporción de examinadores del On-OSCE (97,30%) estaba altamente satisfecha con la puntuación de las habilidades de los estudiantes, mientras que una gran proporción de examinadores del Pa-OSCE (86,67%) reportó bajos niveles de satisfacción. Las Tablas 3 y 4 demuestran que el On-OSCE mejoró la eficiencia y la objetividad de la puntuación, con una alta proporción de examinadores expresando satisfacción con el nuevo sistema de gestión de puntuación. 

**Tabla 5 -:** El nivel de satisfacción del Pa-OSCE y del On-OSCE utilizando la prueba exacta de Fisher. Bantul, Yogyakarta, Indonesia, 2019-2021

**Nivel de satisfacción (puntuaciones totales)**	Pa-OSCE* N (%)	**On-OSCE** ^†^ **N (%)**	**Valor de p/V** ^‡^ **de Cramer**
Alta satisfacción (141 - 200)	4 (13,33)	36 (97,30)	< 0,001/0,85
Satisfacción baja a media (0 - 140)	26 (86,67)	1 (2,70)	
Total	30 (100)	37 (100)	

*Pa-OSCE: Grupo del sistema de gestión de puntuación del OSCE (examen clínico estructurado objetivo) basado en papel;

**^†^ On-OSCE:** Grupo del sistema de gestión de puntuación del OSCE (examen clínico estructurado objetivo) basado en línea;

**^‡^ V de Cramer:** Análisis de SPSS para obtener la asociación entre las variables (Pa-OSCE y On-OSCE)

## Discusión

 El objetivo de este estudio es evaluar de manera integral la satisfacción de los examinadores con el sistema de gestión de puntuación On-OSCE, utilizando herramientas validadas desarrolladas mediante análisis factorial. La evaluación se realizó en cuatro aspectos principales (ahorro de tiempo, facilidad de uso, aplicación prospectiva y objetividad) con un inventario de alta validez y confiabilidad. Además, este estudio demostró de manera concluyente que los examinadores lograron ahorrar un tiempo significativo al asignar puntuaciones finales a los estudiantes en el sistema de gestión de puntuación On-OSCE ( Tabla 3 ). Entre los cuatro aspectos principales, el grupo On-OSCE mostró una alta satisfacción ( Tabla 4 ). Los resultados mostraron una alta proporción (97,30%) de examinadores satisfechos con la eficiencia (ahorro de tiempo) y la objetividad de la puntuación, y aceptados para aplicación futura (facilidad de uso y aplicación prospectiva) en comparación con el Pa-OSCE (p < 0,001) ( Tabla 5 ). 

 Uno de los hallazgos más desalentadores en nuestro contexto fue la mejora drástica en el tiempo de finalización de la puntuación, para calcular la puntuación final y decidir si el examinado aprobaba o reprobaba la habilidad que estaba siendo evaluada. El estudio mostró que, al emplear el sistema de gestión de puntuación On-OSCE, los examinadores necesitaron menos tiempo para completar el proceso de puntuación que con el sistema de gestión de puntuación Pa-OSCE ( Tabla 3 ). Nuestra hipótesis fue coherente con varios estudios que indican que el sistema de gestión de puntuación del OSCE en línea redujo el tiempo para obtener el resultado oficial más rápidamente que el sistema tradicional de gestión de puntuación del OSCE ^(^ 22 ^,^ 30 ^)^ . Hay cuatro razones por las cuales el sistema de gestión de puntuación On-OSCE fue más rápido que el sistema de gestión de puntuación Pa-OSCE. En primer lugar, el sistema de gestión de puntuación del On-OSCE calculó automáticamente las puntuaciones de los estudiantes, garantizó el envío de una lista de verificación sin errores, fue fácilmente marcado y no necesitó ser escrito a mano. En segundo lugar, el sistema de gestión de puntuación del OSCE en línea (tableta o PC) permite a los examinadores marcar la lista de verificación fácilmente, sin esfuerzo y de manera continua ^(^ 10 ^)^ . Además, la lista de verificación marcada por los examinadores es procesada automáticamente por el sistema, lo que hace que las puntuaciones de los estudiantes aparezcan de inmediato ^(^ 24 ^)^ . En tercer lugar, el sistema de gestión de puntuación del On-OSCE alerta sobre las notas faltantes. Al igual que un sistema de alarma, el examinador recibe una alerta en la pantalla de la tableta o PC si falta alguna nota ^(^ 24 ^)^ . En cuarto lugar, el sistema de gestión de puntuación On-OSCE ofrece a los examinadores un acceso directo en la lista de verificación ^(^ 7 ^,^ 22 ^)^ . Los examinadores “clickean” fácilmente en la lista de verificación del On-OSCE en lugar de marcarla con un bolígrafo en el sistema de gestión de puntuación del Pa-OSCE. Los examinadores simplemente usan el sistema de gestión de puntuación On-OSCE desplazando o girando la hoja de la lista de verificación en la pantalla ^(^ 30 ^-^ 31 ^)^ . Así, el sistema de gestión de puntuación On-OSCE fue superior al sistema de gestión de puntuación Pa-OSCE en la finalización de las evaluaciones de los estudiantes. Estos hallazgos sugieren que la aplicación sería beneficiosa para los estudiantes de enfermería y para las instituciones de enfermería, ya que nuestros estudios anteriores indicaron que los estudiantes estaban satisfechos con la evaluación mediante el sistema de gestión de puntuación On-OSCE, ya que obtenían sus puntuaciones de manera inmediata y estas eran increíblemente precisas ^(^ 15 ^)^ . Además, evaluamos la preparación de la enseñanza de enfermería en Indonesia para la implementación del sistema de gestión de puntuación On-OSCE y reconocimos que la mayoría de ellos estaba lista para implementar esta aplicación en su institución ^(^ 25 ^)^ . 

 Evaluar la satisfacción de los examinadores con la implementación del sistema de gestión de puntuación del On-OSCE es vital para obtener una comprensión, especialmente de su viabilidad, y proporcionar información fundamental para programas de mejora futuros. Nuestros resultados mostraron que los examinadores estuvieron muy satisfechos con la asignación de las notas de los estudiantes usando el sistema de gestión de notas del On-OSCE (97,30%), en comparación con el sistema de gestión de notas del Pa-OSCE, que presentó una baja satisfacción (86,67%) ( Tabla 5 ). En relación con la utilización de los sistemas de gestión de puntuación del OSCE en línea, los examinadores evaluaron su nivel de satisfacción en cuatro dominios: ahorro de tiempo, facilidad de uso, aplicaciones prospectivas y objetividad ( Tabla 4 ). Otros estudios han encontrado que las herramientas electrónicas facilitaron el análisis de resultados agregados, lo que resultó en un ahorro significativo de tiempo ^(^ 13 ^,^ 30 ^)^ . Del mismo modo, la utilización de nuestro sistema electrónico exclusivo redujo significativamente el tiempo necesario para el análisis de datos, proporcionando más tiempo para la interpretación de los mismos a fin de mejorar el desarrollo curricular y la evaluación del proceso de aprendizaje, lo que puede ser una de las razones por las que este sistema de gestión de puntuación satisfizo a los examinadores. Además, el sistema de gestión de puntuación del On-OSCE se convirtió en una aplicación futura sin esfuerzo para los examinadores, ya que se familiarizaron rápidamente con el sistema; los examinadores informaron estar altamente satisfechos en los cuatro dominios. Sin embargo, los autores afirmaron que los examinadores deben recibir capacitación y práctica adecuadas para sentirse confiados en el uso del sistema de evaluación informatizado ^(^ 24 ^)^ . 

 De acuerdo con los resultados de este estudio, se identificaron tres motivos principales para la satisfacción de los examinadores con el uso del sistema de gestión de puntuación del On-OSCE. Primero, podían firmar o crear comentarios en el formulario de evaluación en cualquier momento, lo que no podían hacer con el sistema de gestión de puntuación en papel. El sistema de gestión de puntuación del OSCE en línea fue diseñado para que los examinadores pudieran proporcionar *feedback* de manera eficaz y adecuado. Este hallazgo está alineado con investigaciones anteriores que indican que el OSCE en línea ofrece un medio viable para evaluar las competencias de los estudiantes y proporcionar *feedback* inmediato sobre las habilidades evaluadas ^(^ 11 ^,^ 22 ^)^ . En segundo lugar, el sistema de gestión de puntuación del On-OSCE desactivó la función de añadir/restar puntuación, facilitando a los examinadores la asignación objetiva de las puntuaciones generales de los estudiantes de acuerdo con su desempeño. La adición o sustracción de puntuaciones de los estudiantes suele ser realizada por los examinadores en el sistema de gestión de puntuación del Pa-OSCE, lo que disminuye la objetividad de la puntuación. En tercer lugar, la Tabla 2 muestra que hay una diferencia estadísticamente significativa en la edad entre los examinadores. Comparando las edades, el grupo del On-OSCE es más joven que el grupo del Pa-OSCE. Esto puede deberse a que los examinadores adultos jóvenes tienen experiencia en competencias digitales, como el uso de computadoras y servicios de Internet ^(^ 32 ^)^ , y están expuestos a mejores avances tecnológicos en comparación con las personas mayores ^(^ 33 ^)^ . Por lo tanto, deben considerarse trabajos futuros, especialmente para analizar los factores que afectan las decisiones de los examinadores en el sistema electrónico de evaluación del OSCE. 

 Durante la pandemia de COVID-19, el sistema de gestión de puntuación del OSCE en línea permitió a los examinadores evaluar las habilidades de los estudiantes sin una evaluación presencial ^(^ 31 ^)^ . Además, un estudio reconoció que la respuesta favorable de los estudiantes al sistema modificado de gestión de puntuación del OSCE en línea respalda una evaluación en línea para mejorar el sistema tradicional de gestión de puntuación del OSCE ^(^ 7 ^)^ . Sin embargo, los estudiantes creían que el sistema de gestión de puntuación del OSCE en línea hacía que los examinadores ignoraran su comportamiento y se concentraran en las laptops o tabletas, lo que podía aumentar la ansiedad de los estudiantes ^(^ 15 ^)^ . Además, el sistema de gestión de puntuación del On-OSCE puede utilizarse junto con el sistema tradicional de gestión de puntuación del OSCE, siempre que las instituciones cuenten con una conexión adecuada a Internet, *hardware* apropiado (tableta o PC), listas de verificación legítimas de habilidades y examinadores debidamente capacitados ^(^ 21 ^)^ . 

 La competencia clínica se examina de manera consistente, integral y organizada en un amplio espectro. El examen requiere un proceso altamente objetivo en el que el sesgo del examinador debe minimizarse y debe demostrarse la discriminación entre los niveles de desempeño de los estudiantes ^(^ 22 ^)^ . La hipótesis fue coherente con estudios que muestran que el sistema de gestión de puntuación del OSCE en línea redujo los problemas administrativos ^(^ 6 ^,^ 30 ^)^ . Además, nuestros resultados, consistentes con el estudio, revelaron que el *software* electrónico ahorró tiempo y eliminó la posibilidad de pérdida de datos ^(^ 23 ^)^ . Los posibles beneficios del *software* electrónico incluyen la capacidad de almacenar y analizar resultados agregados e individuales, además de proporcionar a los estudiantes un *feedback* objetivo inmediato ^(^ 23 ^)^ . 

 Este estudio aún tiene limitaciones. En primer lugar, al ser un estudio en un solo sitio, los resultados pueden no generalizarse a otras instituciones, países o especialidades de salud. En segundo lugar, no evaluamos las percepciones de los examinadores sobre la cantidad de esfuerzo mental requerido. El esfuerzo mental de los examinadores experimentados puede afectar su desempeño al observar y evaluar a los estudiantes, lo que puede influir en su nivel de satisfacción. Por lo tanto, futuros estudios podrían organizarse para investigar el esfuerzo mental de los examinadores en el Pa-OSCE y en el On-OSCE. En tercer lugar, las variables de nuestro estudio y el tamaño de la muestra son limitados y no cumplen con los requisitos estadísticos ni las suposiciones necesarias para realizar la prueba de regresión logística ^(^ 34 ^)^ . Por lo tanto, se necesitan investigaciones futuras para evaluar las variables que pueden contribuir a la satisfacción de los estudiantes y examinadores de manera integral y aumentar el tamaño de las muestras para permitir pruebas de regresión. 

## Conclusión

El sistema de gestión de puntuación del On-OSCE puede aliviar los desafíos encontrados durante el riguroso proceso del OSCE. En comparación con el sistema tradicional de gestión de puntuación del OSCE, el sistema de gestión de puntuación On-OSCE ofrece ventajas como un mayor ahorro de tiempo, facilidad de uso, aplicación prospectiva y objetividad en la evaluación del desempeño de los estudiantes. Los examinadores que están familiarizados con la funcionalidad del sistema demostraron una mayor satisfacción con la función general y el uso del sistema en comparación con el sistema de gestión de puntuación Pa-OSCE. El sistema de gestión de puntuación del On-OSCE aumenta la eficacia en la planificación y la implementación del OSCE. Tiene enormes ventajas y es viable para pruebas de práctica clínica, que podrían emplearse ampliamente en el aprendizaje práctico en dominios médicos conectados en el futuro. Además, otros estudios pueden utilizar el reciente inventario de satisfacción de 20 ítems para evaluar a los educadores de enfermería en todo el mundo.

# Translation: Satisfação dos educadores de enfermagem com o sistema de pontuação do Objective Structured Clinical Examination online *

**DOI:** 10.1590/1518-8345.6816.4345

**Published:** 2024-11-15

**Authors:** Fahni Haris, Ferika Indarwati, Yanuar Primanda, Resti Yulianti Sutrisno, Kellyana Irawati, Yin-Hwa Shih

**Affiliations:** ^1^ Universitas Muhammadiyah Yogyakarta, School of Nursing, Bantul, Yogyakarta, Indonesia.; ^2^ Asia University, Departement of Healthcare Administration, Wufeng, Taichung, Taiwan.; ^3^ Queensland University of Technology, School of Nursing, Brisbane, Queensland, Australia.

**Keywords:** Educação em Enfermagem, Tecnologia Educacional, Internet, Satisfação do Participante, Questionários, Países em Desenvolvimento

## Abstract

o sistema de pontuação do OSCE em papel (Pa-OSCE) tem várias desvantagens, incluindo o desperdício significativo de papel e o fato de consumir muito tempo. Este estudo tem como objetivo avaliar a satisfação dos examinadores com o sistema de gerenciamento de pontuação do On-OSCE, identificar e avaliar os possíveis benefícios da inovação e promover seu uso.

usando um projeto de estudo transversal, desenvolvemos um inventário de satisfação que consiste em quatro domínios: economia de tempo, facilidade de uso, aplicação prospectiva e objetividade. Esse inventário foi usado para comparar os escores de satisfação entre dois grupos de examinadores (total de 67 indivíduos).

o inventário de satisfação de 20 itens demonstrou alta validade e confiabilidade (0,98 e 0,97, respectivamente). O tempo médio de pontuação foi significativamente menor no grupo do sistema de gerenciamento de pontuação *online* (p<0,001). Tanto a pontuação total de satisfação quanto as pontuações de cada domínio foram significativamente maiores no grupo do sistema de gerenciamento de pontuação *online* em comparação com o grupo do sistema de gerenciamento de pontuação em papel (p<0,001).

uma grande proporção dos examinadores forneceu *feedback* positivo sobre o sistema de gerenciamento de pontuação *online* . O sistema de gerenciamento de pontuação *online* economiza o tempo de pontuação, é mais objetivo, mais fácil de usar e é recomendado para implementação. Os sistemas de gerenciamento de pontuação *online* podem ser implementados em instituições de ensino de enfermagem em todo o mundo para melhorar a eficiência da pontuação. Além disso, o inventário de satisfação de 20 itens pode servir como uma ferramenta de *benchmarking* para avaliar os educadores em todo o mundo.

## Destaques

**(1)** Perguntas de 20 itens válidas e confiáveis sobre o sistema de pontuação de enfermagem.

**(2)** Uma alta proporção de examinadores (professores) forneceu *feedback* positivo sobre o OSCE *online* .

**(3)** As instituições de ensino de enfermagem podem adotar o sistema de pontuação do On-OSCE para melhorar a pontuação.

**(4)** A utilização do On-OSCE pode economizar tempo, ser objetiva e simplificar o processo de pontuação.

**(5)** O sistema de pontuação do On-OSCE é altamente recomendado para implementação em todo o mundo.

## Introdução

 O exame clínico estruturado objetivo (OSCE) foi desenvolvido no Reino Unido para avaliar as competências clínicas dos estudantes de medicina em um ambiente controlado e simulado ^(^ 1 ^)^ . O OSCE foi introduzido e utilizado para avaliar as competências clínicas dos alunos em vários programas de educação profissional na área da saúde, incluindo enfermagem ^(^ 2 ^-^ 4 ^)^ . Há vários métodos de avaliação de competências clínicas na educação de profissionais de saúde, como o miniexercício de avaliação clínica (mini-CEX) e a observação direta de habilidades procedimentais (DOPS); no entanto, o OSCE continua sendo o método mais usado usado para exames de qualificação de profissionais de saúde, inclusive em países asiáticos como a Indonésia ^(^ 5 ^-^ 7 ^)^ . 

 O exame OSCE tem várias estações de teste com ambientes simulados ^(^ 7 ^-^ 9 ^)^ e, durante o exame, os alunos são monitorados e avaliados por um examinador usando critérios de marcação predeterminados ^(^ 10 ^)^ . O OSCE reduz o viés do examinador, padroniza o procedimento do exame e proporciona objetividade para avaliar as competências clínicas ^(^ 1 ^)^ . Além disso, o OSCE destaca os pontos fortes e fracos dos alunos ^(^ 11 ^-^ 13 ^)^ , avalia seus conhecimentos e atitudes em relação à prática clínica ^(^ 14 ^)^ , proporciona um aprendizado profundo ^(^ 4 ^)^ e aumenta a autoconfiança dos alunos ^(^ 3 ^,^ 5 ^)^ . Tanto os alunos quanto os examinadores reconheceram a satisfação e os benefícios da experiência do OSCE em sua formação ^(^ 2 ^,^ 15 ^-^ 17 ^)^ . Além disso, o OSCE tem recebido excelente feedback como ferramenta de avaliação da competência clínica e da diversidade estudantil na educação ^(^ 12 ^,^ 18 ^)^ . É interessante notar que há limitações significativas associadas aos OSCEs. Alguns alunos os consideram estressantes e eles exigem recursos consideráveis, inclusive laboratórios de habilidades clínicas, equipamentos e examinadores ^(^ 13 ^)^ . Apesar dos desafios, autores acreditam que os benefícios educacionais dos OSCEs superam significativamente as preocupações relacionadas à alocação de recursos ^(^ 16 ^)^ . 

 O sistema de gerenciamento de pontuação OSCE em profissões de saúde e educação em enfermagem tem sido tradicionalmente conduzido usando um método baseado em papel (Pa-OSCE). No entanto, os examinadores relataram vários problemas com o sistema Pa-OSCE, incluindo perda de folhas de avaliação, falta de detalhes, como números e nomes dos alunos, e caligrafia ilegível ^(^ 19 ^)^ . Além disso, as pontuações do OSCE não ficam imediatamente disponíveis, o que gera pressão de tempo e reduz a eficiência dos recursos e do gerenciamento dos examinadores do OSCE ^(^ 20 ^)^ . Nosso estudo anterior confirmou as preocupações associadas ao Pa-OSCE, incluindo o desperdício significativo de papel, o tempo excessivo gasto pelos examinadores para calcular manualmente a pontuação com base na lista de verificação e a ausência de uma coluna de *feedback* apropriada na lista de verificação para avaliar o desempenho do examinando ^(^ 21 ^)^ . Portanto, o desenvolvimento de um sistema de gerenciamento de pontuação do OSCE *online* (On-OSCE) é essencial, especialmente nas instituições de ensino de enfermagem da Indonésia, para resolver essas lacunas no Pa-OSCE ^(^ 22 ^)^ . 

 Vários estudos avaliaram a satisfação dos avaliadores com a implementação do sistema de gerenciamento de pontuação do OSCE *online* ^(^ 13 ^,^ 16 ^,^ 23 ^)^ . Esses estudos exploraram as percepções e atitudes dos examinadores em relação ao sistema de gerenciamento de pontuação *online* no ensino de enfermagem na Irlanda ^(^ 23 ^)^ . Os resultados indicaram altos níveis de satisfação dos examinadores. O sistema de gerenciamento de pontuação do OSCE *online* reduziu a falta de dados e melhorou o fornecimento de *feedback* oportuno aos alunos ^(^ 23 ^)^ . Da mesma forma, um estudo sobre a avaliação *online* do OSCE em instituições de ensino de farmácia em Taiwan relatou que os examinadores demonstraram alto reconhecimento, aceitação e satisfação com o sistema de avaliação *online* ^(^ 24 ^)^ . Além disso, o sistema de gerenciamento de pontuação do OSCE *online* facilita o armazenamento de dados e permite a análise detalhada do desempenho geral do grupo ou de cada aluno, o que contribui para uma avaliação mais aprofundada do ensino e da aprendizagem ^(^ 23 ^)^ . 

 Embora o sistema de gerenciamento de pontuação do OSCE *online* esteja bem estabelecido em países desenvolvidos ^(^ 3 ^,^ 6 ^,^ 23 ^)^ , sua implementação em países em desenvolvimento, como a Indonésia, representa uma nova abordagem, especialmente no ensino de enfermagem. Nosso estudo anterior revelou que o sistema comum de gerenciamento de pontuação do OSCE na Indonésia se baseava no sistema tradicional de gerenciamento de pontuação baseado em papel (Pa-OSCE) ^(^ 25 ^)^ . Para resolver problemas como o desperdício excessivo de papel e o consumo de tempo, nosso departamento de enfermagem desenvolveu um sistema de pontuação *online* (On-OSCE) ^(^ 21 ^)^ . Para garantir a eficácia e a aceitação do On-OSCE entre os administradores e examinadores acostumados com técnicas de avaliação baseadas em papel, é fundamental avaliar seu uso prático. Assim, este estudo tem como objetivo avaliar a satisfação dos examinadores com o sistema de gerenciamento de pontuação do On-OSCE, identificar e ponderar os possíveis benefícios dessa inovação e promover sua adoção. 

## Métodos

### Desenho do estudo

 Este estudo utilizou uma abordagem de corte transversal, em que o pesquisador mede simultaneamente os resultados e as exposições dos participantes. O estudo foi realizado na Escola de Enfermagem da *Universitas Muhammadiyah Yogyakarta* , Indonésia, de 21 de junho de 2019 a 28 de fevereiro de 2021. A coleta de dados se estendeu por três anos para apoiar a transição de um OSCE em papel para um sistema de gerenciamento de pontuação de OSCE *online* e para permitir a captura de dados de ambos os grupos (em papel vs. *online* ). 

### Participantes

 Usamos uma técnica de amostragem total neste estudo. Um total de 30 examinadores usando um sistema de gerenciamento de pontuação de OSCE em papel (Pa-OSCE) e 37 examinadores usando um sistema de gerenciamento de pontuação de OSCE *online* (On-OSCE) participaram do estudo. Os examinadores eram professores de enfermagem ou assistentes de professores da Escola de Enfermagem da *Universitas Muhammadiyah Yogyakarta* , Indonésia, que realizam regularmente avaliações OSCE na escola de enfermagem. 

### Instrumentos

 Antes de coletar os dados, desenvolvemos um inventário de satisfação com 20 itens abrangendo quatro domínios: economia de tempo (6 itens), facilidade de uso (5 itens), aplicação prospectiva (4 itens) e objetividade (5 itens), conforme detalhado na Tabela 1 . Os participantes foram solicitados a classificar sua satisfação com cada item em uma escala do tipo Likert de 11 pontos, variando de insatisfeito (0) a altamente satisfeito (10) ^(^ 26 ^)^ . O instrumento foi desenvolvido por pesquisadores com base na revisão da literatura sobre esse tópico. Classificamos a satisfação dos participantes em três grupos com base em sua pontuação total: 0-70 indicava baixa satisfação, 71-140 indicava média satisfação e 141-200 indicava alta satisfação. 

 Recrutamos cinco membros do corpo docente com experiência em Pa-OSCE e On-OSCE para realizar um índice de validade de conteúdo do inventário (IVC). O IVC consiste em conteúdo adequado (IVCs) e precisão de palavras (IVCw) ^(^ 27 ^)^ . As pontuações do IVC de cinco especialistas foram de 0,98. Três ou mais especialistas com um CVI médio de 0,78 ou mais são considerados como tendo boa validade de conteúdo ^(^ 28 ^)^ . Também examinamos o valor da correlação item-total nos quatro domínios (um total de 20 questões) para avaliar a satisfação dos examinadores com o uso do Pa-OSCE e do sistema de gerenciamento de pontuação On-OSCE. Os itens com um valor de correlação maior que 0,3 foram mantidos. A Confiabilidade Composta (CC) para o extremo foi estatisticamente significativa (p < 0,05). 

**Tabela 1 -:** A análise de itens do inventário de satisfação. Bantul, Yogyakarta, Indonésia, 2019-2021

**Pergunta**	**Item**	Grupo extremo Valor CC*	**Correlação item-total**	**Nota**
1	Economia de tempo	14,11 ^†^	0,97	manter
2		19,95 ^†^	0,96	manter
3		17,71 ^†^	0,97	manter
4		20,11 ^†^	0,96	manter
5		23,90 ^†^	0,98	manter
6		13,47 ^†^	0,96	manter
1	Fácil de usar	6,66 ^†^	0,92	manter
2		8,84 ^†^	0,95	manter
3		9,36 ^†^	0,95	manter
4		12,25 ^†^	0,94	manter
5		9,99 ^†^	0,87	manter
1	Aplicação prospectiva	7,71 ^†^	0,97	manter
2		8,01 ^†^	0,97	manter
3		7,42 ^†^	0,92	manter
4		9,44 ^†^	0,91	manter
1	Objetividade	7,64 ^†^	0,71	manter
2		10,03 ^†^	0,80	manter
3		6,71 ^†^	0,72	manter
4		3,17 ^†^	0,39	manter
5		10,13 ^†^	0,58	manter

*CC Confiabilidade composta

^†^ Valor significativo p < 0,001

 A confiabilidade (α de Cronbach) e a consistência interna (comparações de grupos extremos e correlação item-total corrigida) foram analisadas usando os dados de 15 examinadores que conduziram o sistema de gerenciamento de pontuação Pa-OSCE e 15 examinadores que conduziram o sistema de gerenciamento de pontuação On-OSCE. O escore α de Cronbach foi de 0,97. Com um alfa > 0,60, o questionário pode ser considerado confiável ^(^ 26 ^,^ 29 ^)^ . 

### Coleta de dados

 Os convites para a pesquisa foram enviados via aplicativo *WhatsApp* a todos os participantes elegíveis. Os participantes que se mostraram dispostos a participar do estudo podiam entrar em contato com os pesquisadores se precisassem de mais informações relacionadas à pesquisa. A pesquisa de satisfação dos examinadores foi administrada *online* usando o *Google Forms* . Os formulários incluíam 20 perguntas que abrangiam quatro variáveis (economia de tempo, facilidade de uso, aplicação prospectiva e objetividade). O preenchimento e o envio dos formulários indicaram o consentimento dos participantes em participar desse estudo. 

### Análise de dados

 Os dados quantitativos obtidos do desempenho dos examinadores nas estações de OSCE e da pesquisa por questionário foram analisados descritivamente usando o *Microsoft Excel 365* para *Windows 10* (Microsoft Corporation, Redmond, WA, EUA) e o *software* estatístico *IBM SPSS Statistics* versão 22 (IBM Corporation, Armonk, NY, EUA). Os dados descritivos foram apresentados em frequência e porcentagem para dados categóricos, como gênero, formação e experiência de ensino dos participantes, ou média para variáveis contínuas, como idade dos participantes. A classificação média foi usada para descrever os dados em minutos para concluir a pontuação e as pontuações de satisfação em quatro domínios (economia de tempo, facilidade de uso, aplicação prospectiva e objetividade). 

 Uma análise mais aprofundada foi realizada para verificar as diferenças entre os grupos Pa-OSCE e On-OSCE em termos demográficos, o tempo necessário para concluir as pontuações dos alunos e a satisfação do ponto de vista dos examinadores. Testes apropriados, como os testes Exato de Fisher, Qui-quadrado ou Mann-Whitney U, foram usados para investigar as diferenças médias entre os grupos de pontuação do OSCE em papel e *online* nos quatro domínios de satisfação dos examinadores. Os dados dos escores totais de satisfação dos examinadores foram inicialmente categorizados em três categorias (baixa, média e alta). No entanto, devido às poucas respostas na categoria de baixa satisfação, esse grupo foi mesclado com a categoria de satisfação média para permitir uma análise significativa. A significância estatística foi definida como p < 0,05. 

### Considerações éticas

 A autorização ética foi concedida pelo Comitê de Ética da Faculdade de Medicina e Ciências da Saúde da *Universitas Muhammadiyah Yogyakarta* (nº 051/EC-KEPK FKIK UMY/II/2019). Os participantes foram informados sobre o objetivo do estudo e forneceram consentimento verbal para participar. Foi-lhes assegurado que seus dados permaneceriam confidenciais. As respostas foram mantidas anônimas e um acordo de não divulgação foi estabelecido, dando aos entrevistados o direito de se retirarem do estudo a qualquer momento. 

## Resultados

### Dados demográficos dos participantes

 A Tabela 2 mostra as diferenças estatísticas entre os grupos com base nas categorias de idade, gênero, nível educacional e experiência de ensino. O resultado mostra que as características dos participantes em ambos os grupos eram bastante semelhantes, com exceção da idade. A categoria de idade foi significativamente diferente entre essas quatro variáveis, enquanto o gênero, o nível educacional e a experiência de ensino não foram significativos (p > 0,05). A média de idade dos entrevistados foi de 34,10 ± 5,58 no grupo Pa-OSCE e de 30,70 ± 5,78 no grupo On-OSCE (p = 0,011). A maioria dos examinadores era do sexo feminino, tanto no grupo Pa-OSCE (90%) quanto no grupo On-OSCE (89,19%). A maioria dos examinadores tinha nível de pós-graduação, com 66,67% no grupo Pa-OSCE e 56,76% no grupo On-OSCE. A experiência de ensino dos entrevistados foi categorizada em menos de um ano ou igual a um ano de experiência e menos de dez anos de experiência. 

**Tabela 2 -:** Características demográficas dos entrevistados. Bantul, Yogyakarta, Indonésia, 2019-2021

**Variáveis**	Pa-OSCE* N (%)	**On-OSCE** ^†^ **N (%)**	**Valor de p**
Sexo			1,000 ^‡^
Feminino	27 (90,00)	33 (89,19)	
Masculino	3 (10,00)	4 (10,81)	
Nível educacional			0,458 ^‡^
Graduação	10 (33,33)	16 (43,24)	
Pós-graduação	20 (66,67)	21 (56,76)	
Experiência de ensino			0,732 ^‡^
≤ 1 ano	12 (40,00)	13 (35,14)	
≤ 5 anos	6 (20,00)	12 (32,43)	
≤ 10 anos	10 (33,33)	10 (27,03)	
> 10 anos	2 (6,67)	2 (5,40)	
Idade [Média (SD ^§^ )]	34,10 (5,58)	30,70 (5,78)	0,011 ^||^

*Pa-OSCE: Grupo do sistema de gerenciamento de pontuação do OSCE (exame clínico estruturado objetivo) baseado em papel;

**^†^ On-OSCE:** Grupo do sistema de gerenciamento de pontuação do OSCE (exame clínico estruturado objetivo) baseado em computador;

^‡^ Teste exato de Fisher;

^§^ SD Desvio-padrão;

^||^ Teste de Mann-Whitney

### Os examinadores passaram menos tempo pontuando com o On-OSCE

 Houve uma diferença estatisticamente significativa no tempo necessário para concluir a pontuação do OSCE no Pa-OSCE e no On-OSCE. A classificação média do grupo On-OSCE foi menor do que a do grupo Pa-OSCE, como mostra a Tabela 3 . Os examinadores do Pa-OSCE passaram mais tempo calculando a pontuação, enquanto as pontuações no On-OSCE foram calculadas automaticamente pelo sistema. Os dados revelam que os examinadores economizaram mais tempo ao atribuir a pontuação final ao aluno do grupo On-OSCE. 

**Tabela 3 -:** A classificação média do tempo médio gasto na pontuação entre os dois grupos usando o teste U de Mann-Whitney. Bantul, Yogyakarta, Indonésia, 2019-2021

	Pa-OSCE*	**On-OSCE** ^†^	**Valor de p**
Tempo gasto na pontuação	49,58	21,36	< 0,001

*Pa-OSCE: Grupo do sistema de gerenciamento de pontuação do OSCE (exame clínico estruturado objetivo) baseado em papel;

**^†^ On-OSCE:** Grupo do sistema de gerenciamento de pontuação do OSCE (exame clínico estruturado objetivo) baseado em computador

###  Os examinadores tiveram um *feedback* positivo sobre o sistema de gerenciamento de pontuação On-OSCE 

 Os escores de satisfação em quatro domínios do inventário são mostrados na Tabela 4 . Foi registrada uma diferença estatisticamente significativa entre os grupos. Os resultados indicaram que o On-OSCE ofereceu economia de tempo, facilidade de uso, aplicações prospectivas e objetividade em comparação com o Pa-OSCE. O On-OSCE melhorou a eficiência da pontuação (economia de tempo) e a objetividade e foi aceito (fácil de usar) para uso futuro (aplicações prospectivas) pela maioria dos examinadores. 

**Tabela 4 -:** A classificação média dos escores de satisfação em quatro domínios usando o teste U de Mann-Whitney. Bantul, Yogyakarta, Indonésia, 2019-2021

**Variáveis**	Pa-OSCE*	**On-OSCE** ^†^	**Valor de p**
Economia de tempo (0 - 60)	16,30	48,35	<0,001
Fácil de usar (0 - 50)	18,48	46,58	<0,001
Aplicação prospectiva (0 - 40)	15,62	48,91	<0,001
Objetividade (0 - 50)	22,80	43,08	<0,001
Índice de satisfação total	15,82	48,74	<0,001

*Pa-OSCE: Grupo do sistema de gerenciamento de pontuação do OSCE (exame clínico estruturado objetivo) baseado em papel;

**^†^ On-OSCE:** Grupo do sistema de gerenciamento de pontuação do OSCE (exame clínico estruturado objetivo) baseado em computador

### A satisfação dos examinadores foi maior no grupo On-OSCE em comparação com o grupo Pa-OSCE

 Combinamos as categorias de escores de satisfação total baixos e médios por motivos estatísticos. A satisfação dos examinadores com o Pa-OSCE e o On-OSCE é mostrada na Tabela 5 . Uma grande proporção de examinadores do On-OSCE (97,30%) estava altamente satisfeita com a pontuação das habilidades dos alunos, enquanto uma grande proporção de examinadores do Pa-OSCE (86,67%) relatou baixos níveis de satisfação. As Tabelas 3 e 4 demonstram que o On-OSCE melhorou a eficiência e a objetividade da pontuação, com uma alta proporção de examinadores expressando satisfação com o novo sistema de gerenciamento de pontuação. 

**Tabela 5 -:** O nível de satisfação do Pa-OSCE e do On-OSCE usando o teste exato de Fisher. Bantul, Yogyakarta, Indonésia, 2019-2021

**Nível de satisfação (escores totais)**	Pa-OSCE N (%)	**On-OSCE** ^†^ **N (%)**	**Valor de p /V** ^‡^ **de Cramer**
Alta satisfação (141 - 200)	4 (13,33)	36 (97,30)	< 0,001/0,85
Satisfação baixa a média (0 - 140)	26 (86,67)	1 (2,70)	
Total	30 (100)	37 (100)	

*Pa-OSCE: Grupo do sistema de gerenciamento de pontuação do OSCE (exame clínico estruturado objetivo) baseado em papel;

**^†^ On-OSCE:** Grupo do sistema de gerenciamento de pontuação do OSCE (exame clínico estruturado objetivo) baseado em computador;

**^‡^ V de Cramer:** Análise SPSS para obter a associação entre as variáveis (Pa-OSCE e On-OSCE)

## Discussão

 O objetivo deste estudo é avaliar de forma abrangente a satisfação dos examinadores com o sistema de gerenciamento de pontuação On-OSCE, utilizando ferramentas validadas desenvolvidas por meio de análise fatorial. A avaliação foi realizada em quatro aspectos principais (economia de tempo, facilidade de uso, aplicação prospectiva e objetivo) com um inventário de alta validade e confiabilidade. Além disso, esse estudo demonstrou de forma conclusiva que os examinadores conseguiram economizar um tempo significativo ao atribuir pontuações finais aos alunos no sistema de gerenciamento de pontuação On-OSCE ( Tabela 3 ). Entre os quatro aspectos principais, o grupo do On-OSCE demonstrou alta satisfação ( Tabela 4 ). Os resultados mostraram uma alta proporção (97,30%) de examinadores satisfeitos com a eficiência (economia de tempo) e objetividade da pontuação e aceitos para aplicação futura (facilidade de uso e aplicação prospectiva) em comparação com o Pa-OSCE (p < 0,001) ( Tabela 5 ). 

 Uma das descobertas mais desanimadoras em nosso contexto foi a melhora drástica no tempo de finalização da pontuação, para calcular a pontuação final e decidir se o examinando foi aprovado ou reprovado na habilidade que estava sendo examinada. O estudo mostrou que, ao empregar o sistema de gerenciamento de pontuação On-OSCE, os examinadores precisaram de menos tempo para concluir o processo de pontuação do que o sistema de gerenciamento de pontuação Pa-OSCE ( Tabela 3 ). Nossa hipótese foi coerente com vários estudos que indicam que o sistema de gerenciamento de pontuação do OSCE *online* reduziu o resultado oficial mais rapidamente do que o sistema tradicional de gerenciamento de pontuação do OSCE ^(^ 22 ^,^ 30 ^)^ . Há quatro razões pelas quais o sistema de gerenciamento de pontuação On-OSCE foi mais rápido do que o sistema de gerenciamento de pontuação Pa-OSCE. Em primeiro lugar, o sistema de gerenciamento de pontuação do On-OSCE calculou as pontuações dos alunos automaticamente, garantiu o envio de uma lista de verificação sem erros, foi facilmente marcado e não precisou ser escrito à mão. Em segundo lugar, o sistema de gerenciamento de pontuação do OSCE *online* ( *tablet* ou PC) permite que os examinadores marquem a lista de verificação com facilidade, sem esforço e continuamente ^(^ 10 ^)^ . Além disso, a lista de verificação marcada pelos examinadores é processada automaticamente pelo sistema, fazendo com que as pontuações dos alunos apareçam imediatamente ^(^ 24 ^)^ . Em terceiro lugar, o sistema de gerenciamento de pontuação do On-OSCE avisa sobre as notas perdidas pelos sistemas. Assim como no sistema de alarme, o examinador recebe um alerta na tela do *tablet* ou do PC se uma nota estiver faltando ^(^ 24 ^)^ . Em quarto lugar, o sistema de gerenciamento de pontuação On-OSCE oferece aos examinadores um ponto direto na lista de verificação ^(^ 7 ^,^ 22 ^)^ . Os examinadores “clicam” facilmente na lista de verificação do On-OSCE em vez de marcá-la com caneta no sistema de gerenciamento de pontuação do Pa-OSCE. Os examinadores simplesmente usam o sistema de gerenciamento de pontuação On-OSCE virando a folha da lista de verificação, arrastando ou rolando a lista de verificação na tela ^(^ 30 ^-^ 31 ^)^ . Assim, o sistema de gerenciamento de pontuação On-OSCE foi superior ao sistema de gerenciamento de pontuação Pa-OSCE na conclusão das avaliações dos alunos. Esses achados acreditam que o aplicativo seria benéfico para os estudantes de enfermagem e para as instituições de enfermagem, pois nossos estudos anteriores afirmaram que os estudantes estavam satisfeitos com a avaliação pelo sistema de gerenciamento de pontuação On-OSCE, pois recuperavam suas pontuações imediatamente e as pontuações eram incrivelmente precisas ^(^ 15 ^)^ . Além disso, avaliamos a prontidão do ensino de enfermagem na Indonésia para a implementação do sistema de gerenciamento de pontuação On-OSCE e, portanto, reconhecemos que a maioria deles estava pronta para implementar esse aplicativo em sua instituição ^(^ 25 ^)^ . 

 Avaliar a satisfação dos examinadores com a implementação do sistema de gerenciamento de pontuação do On-OSCE é vital para obter uma compreensão, especialmente de sua viabilidade, e fornecer informações fundamentais para programas de aprimoramento futuros. Nossos resultados mostraram que os examinadores ficaram muito satisfeitos com a atribuição das notas dos alunos usando o sistema de gerenciamento de notas do On-OSCE (97,30%), em comparação com o sistema de gerenciamento de notas do Pa-OSCE, que apresentou baixa satisfação (86,67%) ( Tabela 5 ). Com relação à utilização dos sistemas de gerenciamento de pontuação do OSCE *online* , os examinadores avaliaram seu nível de satisfação em quatro domínios: economia de tempo, facilidade de uso, aplicações prospectivas e objetivos ( Tabela 4 ). Outros estudos descobriram que as ferramentas eletrônicas facilitaram a análise de resultados agregados, resultando em economia significativa de tempo ^(^ 13 ^,^ 30 ^)^ . Da mesma forma, a utilização do nosso sistema eletrônico exclusivo reduziu significativamente o tempo necessário para a análise dos dados, proporcionando mais tempo para a interpretação dos dados a fim de melhorar o desenvolvimento do currículo e a avaliação do processo de aprendizagem, o que pode ser um dos motivos pelos quais esse sistema de gerenciamento de pontuação satisfez os examinadores. Além disso, o sistema de gerenciamento de pontuação do On-OSCE tornou-se uma aplicação futura sem esforço para os examinadores, pois eles foram rapidamente reconhecidos e se familiarizaram com o sistema; os examinadores relataram estar altamente satisfeitos em todos os quatro domínios. No entanto, os autores afirmaram que os examinadores devem receber treinamento e prática adequados para se sentirem confiantes no uso do sistema de avaliação computadorizado ^(^ 24 ^)^ . 

 De acordo com os resultados desse estudo, percebeu-se que havia três motivos principais para a satisfação dos examinadores com o uso do sistema de gerenciamento de pontuação do On-OSCE. Primeiro, eles podiam assinar ou criar comentários no formulário de avaliação a qualquer momento, o que não podiam fazer com o sistema de gerenciamento de pontuação em papel. O sistema de gerenciamento de pontuação do OSCE *online* foi projetado para que os examinadores pudessem fornecer seu *feedback* de forma eficaz e adequada. Esse achado está alinhado com pesquisas anteriores que indicam que o OSCE *online* oferece um meio viável de avaliar as competências dos alunos e fornecer *feedback* imediato sobre as habilidades avaliadas ^(^ 11 ^,^ 22 ^)^ . Em segundo lugar, o sistema de gerenciamento de pontuação do On-OSCE desativou a função de adicionar/subtrair pontuação, facilitando para os examinadores a atribuição objetiva das pontuações gerais dos alunos de acordo com seu desempenho. A adição ou subtração das pontuações dos alunos geralmente é feita pelos examinadores no sistema de gerenciamento de pontuação do Pa-OSCE, o que diminui a objetividade da pontuação. Em terceiro lugar, a Tabela 2 mostra que há uma diferença estatisticamente significativa na idade entre os examinadores. Comparando as idades, o grupo On-OSCE é mais jovem do que o grupo Pa-OSCE. Isso pode ser devido ao fato de os examinadores adultos jovens terem experiência em competências digitais, como o uso de computadores e serviços de Internet ^(^ 32 ^)^ , e estarem expostos a melhores desenvolvimentos tecnológicos em comparação com pessoas mais velhas ^(^ 33 ^)^ . Portanto, trabalhos futuros devem ser considerados, especialmente para analisar os fatores que afetam as decisões dos examinadores no sistema eletrônico de avaliação OSCE. 

 Durante a pandemia da COVID-19, o sistema de gerenciamento de pontuação do OSCE *online* permite que os examinadores examinem as habilidades dos alunos sem avaliação presencial ^(^ 31 ^)^ . Além disso, um estudo reconheceu que a resposta favorável dos alunos ao sistema modificado de gerenciamento de pontuação do OSCE *online* apoia uma avaliação *online* para aprimorar o sistema tradicional de gerenciamento de pontuação do OSCE ^(^ 7 ^)^ . No entanto, os alunos acreditavam que o sistema de gerenciamento de pontuação do OSCE *online* faz com que os examinadores se concentrem nos *laptops* ou *tablets* e ignorem seus comportamentos, o que pode aumentar a ansiedade dos alunos ^(^ 15 ^)^ . Além disso, o sistema de gerenciamento de pontuação On-OSCE pode ser utilizado em conjunto com o sistema tradicional de gerenciamento de pontuação OSCE, desde que as instituições tenham uma conexão adequada com a Internet, *hardware* apropriado ( *tablet* ou PC), listas de verificação de habilidades legítimas e examinadores devidamente treinados ^(^ 21 ^)^ . 

 A proficiência clínica é examinada de forma consistente, abrangente e organizada em um amplo espectro. O exame exige um processo altamente objetivo no qual o viés do examinador deve ser diminuído e a discriminação entre os níveis de desempenho dos alunos deve ser demonstrada ^(^ 22 ^)^ . A hipótese foi coerente com estudos que mostram que o sistema de gerenciamento de pontuação do OSCE *online* reduziu o problema administrativo ^(^ 6 ^,^ 30 ^)^ . Além disso, nossos resultados, consistentes com o estudo, revelaram que o software eletrônico economizou tempo e eliminou a possibilidade de falta de dados ^(^ 23 ^)^ . Além disso, os possíveis benefícios do software eletrônico incluem a capacidade de armazenar e analisar resultados agregados e individuais, além de fornecer aos alunos um *feedback* objetivo imediato ^(^ 23 ^)^ . 

 Este estudo ainda tem limitações. Primeiro, por ser um estudo em um único local, os resultados podem não ser generalizados para outras instituições, nações ou especialidades de saúde. Em segundo lugar, não avaliamos as percepções dos examinadores sobre a quantidade de esforço mental necessário. O esforço mental dos examinadores experientes pode afetar seu desempenho ao observar e avaliar os alunos, o que pode afetar o nível de satisfação dos examinadores. Assim, trabalhos futuros podem ser organizados para investigar o esforço mental dos examinadores no Pa-OSCE e no On-OSCE. Em terceiro lugar, as variáveis do nosso estudo e o tamanho da amostra são limitados e não atendem aos requisitos estatísticos ou às suposições necessárias para executar o teste de regressão logística ^(^ 34 ^)^ . Portanto, pesquisas futuras precisam ser realizadas para avaliar as variáveis que podem contribuir para a satisfação dos alunos e examinadores de forma abrangente e melhorar o tamanho das amostras para permitir testes de regressão. 

## Conclusão

O sistema de gerenciamento de pontuação do On-OSCE pode aliviar os desafios encontrados durante o rigoroso processo OSCE. Em comparação com o sistema tradicional de gerenciamento de pontuação do OSCE, o sistema de gerenciamento de pontuação On-OSCE oferece as vantagens de maior economia de tempo, facilidade de uso, aplicação prospectiva e objetividade na avaliação do desempenho dos alunos. Os examinadores que estão familiarizados com a funcionalidade do sistema demonstraram maior satisfação com a função geral e a utilização do sistema do que o sistema de gerenciamento de pontuação Pa-OSCE. O sistema de gerenciamento de pontuação do On-OSCE aumenta a eficácia do planejamento e da implementação do OSCE. Ele tem enormes vantagens e é viável para testes de prática clínica, que poderão ser amplamente empregados no aprendizado prático em domínios médicos conectados no futuro. Além disso, outros estudos podem usar o recente inventário de satisfação de 20 itens para avaliar os educadores de enfermagem em todo o mundo.
